# White Matter Hyperintensities in Parkinson’s Disease: Do They Explain the Disparity between the Postural Instability Gait Difficulty and Tremor Dominant Subtypes?

**DOI:** 10.1371/journal.pone.0055193

**Published:** 2013-01-31

**Authors:** Talia Herman, Keren Rosenberg-Katz, Yael Jacob, Eitan Auriel, Tanya Gurevich, Nir Giladi, Jeffrey M. Hausdorff

**Affiliations:** 1 Movement Disorders Unit, Department of Neurology, Tel-Aviv Sourasky Medical Center, Tel-Aviv, Israel; 2 Functional Brain Imaging Unit, Wohl Institute for Advanced Imaging, Tel-Aviv Sourasky Medical Center, Tel-Aviv, Israel; 3 Sackler Faculty of Medicine, Tel-Aviv University, Tel-Aviv, Israel; 4 Department of Medicine, Harvard Medical School, Boston, Massachusetts, United States of America; Oslo University Hospital, Norway

## Abstract

**Background:**

Brain white matter hyperintensities (WMHs) commonly observed on brain imaging of older adults are associated with balance and gait impairment and have also been linked to cognitive deficits. Parkinson’s disease (PD) is traditionally sub-classified into the postural instability gait difficulty (PIGD) sub-type, and the tremor dominant (TD) sub-type. Considering the known association between WMHs and axial symptoms like gait disturbances and postural instability, one can hypothesize that WMHs might contribute to the disparate clinical sub-types of patients with PD.

**Methods:**

110 patients with PD underwent a clinical evaluation and a 3T MRI exam. Based on the Unified Parkinson Disease Rating Scale, the patients were classified into motor sub-types, i.e., TD or PIGD, and scores reflecting PIGD and TD symptoms were computed. We compared white matter burden using three previously validated methods: one using a semi-quantitative visual rating scale in specific brain regions and two automated methods.

**Results:**

Overall, MRI data were obtained in 104 patients. The mean WMHs scores and the percent of subjects with lesions in specific brain regions were similar in the two subtypes, p = 0.678. The PIGD and the TD scores did not differ even when comparing patients with a relatively high burden of WMHs to patients with a relatively low burden. Across most of the brain regions, mild to moderate correlations between WMHs and age were found (r = 0.23 to 0.41; p<0.021). Conversely, no significant correlations were found between WMHs and the PIGD score or disease duration. In addition, depressive symptoms and cerebro-vascular risk factors were similar among the two subtypes.

**Conclusions:**

In contrast to what has been reported previously among older adults, the present study could not demonstrate any association between WMHs and the PIGD or TD motor sub-types in patients with PD.

## Introduction

Parkinson’s disease (PD) is a progressive neurodegenerative disorder, manifested by a broad spectrum of motor and non-motor features [1]. PD may have variable expressions [2] with differences in the disease profile and progression rate [3]. This clinical heterogeneity in PD is reflected as clinical subtypes. In fact, classification according to the disease’s key motor signs into the Postural Instability Gait Difficulty (PIGD) and tremor dominant (TD) subtypes is often used to distinguish patients who have very different clinical symptoms [4].

White matter hyperintensities (WMHs), also referred to as leukoaraiosis, are commonly observed on brain imaging studies in older adults, often presenting as signal hyperintensities in MRI images [5]. These WMHs are associated with and apparently contribute to balance and gait impairment in aging populations[6]–[14]. Given that some of the motor changes that have been associated with WMHs overlap with features of PD, one can hypothesize that comorbid WM lesions might contribute to the clinical symptoms of PD and might explain some of the differences between PD patients with PIGD symptoms, as compared to TD patients [15]. WMHs have also been linked with general cognitive decline in older adults and in normal aging[16]–[18], and with impairment in attention, executive functions and processing speed [19], [20]. Interestingly, these cognitive changes are commonly observed among patients with PD, even in the early stages of the disease [21]–[24].

Among patients with PD, WMHs may contribute to the development of dementia due, in part, to micro-vascular changes[25]–[30]. Even in the absence of dementia, cognitive deficits are common in patients with PD [31], [32], especially among patients with the PIGD subtype. Patients with vascular parkinsonism (VaPD), high level gait disorders (HLGD), and multiple system atrophy–parkinsonian type (MSA-P) all have balance and gait disturbances similar to those seen in the PIGD subtype[33]–[38]. Moreover, these symptoms are typically accompanied by impaired executive function [34] and urinary incontinence, two symptoms that both may reflect frontal lobe dysfunction[39]–[41].

Studies of leuckoaraiosis severity in PD and its association with motor impairment show variable results[42]–[46]. A few reports suggest that WM lesions are more common in patients with PD compared to normal elderly individuals [47], [48], while other studies failed to demonstrate this relationship [49], [50]. Only two previous studies directly investigated the role of leuckoaraiosis and its relationship to the clinical motor phenotypes of PD. Lee et al. [51]compared 75 TD patients to 54 PIGD and found that WMHs correlated mildly with bradykinesia and rigidity, but not with tremor. Recently, Bohnen and colleagues [52] evaluated 25 TD and 36 PIGD patients; they concluded that WM burden was higher in the PIGD subtype compared with the TD. Leukoaraiosis was significantly correlated with posture, postural stability and with self-report of walking difficulties. Somewhat surprisingly, two of the main PIGD symptoms, freezing of gait and falls were not significantly related to WM findings.

The aim of the present study was, therefore, to compare brain WM burden in PD motor subtypes and to evaluate the associations between different PIGD-related symptoms and MRI findings.

## Materials and Methods

### Experimental Design

The study was conducted in the Laboratory for Gait and Neurodynamics, the Movement Disorders Unit, in the Department of Neurology at the Tel Aviv Sourasky Medical Center. Informed written consent was obtained and the study was conducted according to the principles expressed in the Declaration of Helsinki, as approved by the human studies committee of the Tel Aviv Sourasky Medical Center. In this case-control study, patients were studied on two separate occasions. The first visit included a neurological and clinical examination. Several tests were first performed in the practically-defined “off” medication state (i.e., at least 12 hours after intake of anti-parkinsonian medications) to reflect the underlying pathological state. Subsequently, patients took their routine morning medications and the evaluation continued when the patients reached their “on” state; tests in this condition reflect typical everyday performance. The MRI scan was performed on a separate visit that took place within 2 weeks of the clinical exam.

### Study Participants

One-hundred and ten patients with idiopathic PD were recruited from our databases, referrals from specialists at the outpatient movement disorders unit, and from other affiliated clinics. Subjects were included if they were diagnosed by a movement disorders specialist as having idiopathic PD (as defined by the UK Brain Bank criteria [53]), were between 40 and 85 of age, and were not demented. Subjects were excluded if they had brain surgery in the past including implanted deep brain stimulation or had significant co-morbidities likely to affect gait, e.g., acute illness, orthopedic disease, or history of stroke. In addition, subjects who could not walk independently in the off medication cycle, patients with claustrophobia, and patients who could not undergo MRI testing (e.g., if they had large metal implants) were excluded.

### Classification into PIGD and TD Subtypes

Initially, patients were classified into PIGD, TD or indeterminate groups using the approach proposed in 1990 by Jankovic et al. [54]. Based on the original Unified Parkinson’s Disease Rating Scale (UPDRS) [55], we computed a mean tremor score of 9 tremor items (right and left arm tremor by history, lips or chin tremor, tremor in all 4 limbs, and both arms action or postural tremor on examination) as well as a mean score of 5 PIGD items (i.e., falling, freezing, and walking difficulty by history, gait and postural instability on examination) [56]. Patients were assigned to the tremor group (TD) if the ratio of the mean tremor score divided by the mean PIGD score was greater than or equal to 1.5 [57]. Patients were assigned to the PIGD group if this ratio was equal to or less than 1.0 [58]. When the tremor/PIGD ratio was more than 1.0 and less than 1.5, patients were classified as undetermined [59]. In addition, in order to assess correlations between motor symptoms and WMHs, we used a total TD and PIGD score rather than the ratio (i.e., the sum of 5 items for the PIGD score, and the 9 items for the tremor score) [60]. To further investigate possible associations between WMHs and motor symptoms, in addition to the total PIGD and TD scores, we also computed three additional continuous measures derived from the UPDRS parts II and III: 1) a gait sub-score, 2) a sub-score comprised from last five measures of the motor part, and 3) the latter score with the addition of the FOG item.

After performing the ratio based classification, we found that some of the patients still showed mixed PD symptoms. For example, patients assigned to the TD group had extensive freezing of gait and patients from the PIGD group had severe tremor (see Table 1). Therefore, a stricter criterion was applied on top of the Jankovic based classification to identify two representative subtypes, with minimal symptom overlap, referred to here as predominantly-PIGD (p-PIGD) and predominantly-TD (p-TD). To compare WMHs in patients with minimal overlapping of motor symptoms, patients were excluded from the TD group if they had a PIGD score higher than 3 or a tremor score lower than 4. Similarly, patients were excluded from the PIGD group if their tremor score was higher than 3 or their PIGD score was lower than 4.

**Table 1:** Demographics and basic disease characteristics.

Measure	All Patients (n = 110)	PIGD Patients(n = 62)	TD Patients(n = 42)	Undetermined(n = 6)	P-value(PIGD vs. TD)
Age (yrs)	65.13**±**9.23	64.80**±**7.96	65.88±11.00	63.33±8.98	0.587
Gender (% male)	75.5%	74.2%	81.0%	50.0%	0.242
Education (yrs)	15.47±3.74	15.85±3.98	15.17±3.38	13.67±3.39	0.361
Weight (kg)	77.54**±**12.40	78.11**±**13.65	77.58**±**12.4	70.95±10.67	0.965
Height (cm)	169.3±8.7	169.0±8.4	170.7±8.7	164.33±8.9	0.258
Body-mass-index (kg/m2)	26.85±3.52	27.14±3.72	26.41±3.24	26.23±3.20	0.322
Disease duration (yrs)	5.58±3.52	5.59±3.8	5.38±2.98	7.33±4.33	0.691
UPDRS motor score “off”	40.45±13.03	38.11±11.30	42.79±14.16	48.33±17.69	0.065
UPDRS motor score “on”	33.85±11.96	32.65±11.13	35.79±13.32	32.83±10.14	0.195
Total UPDRS score “on”	60.69±19.61	60.19±17.62	60.50±23.11	67.16±12.76	0.942
Hoehn & Yahr stage “off”	2.58±0.69	2.83±0.75	2.24±0.43	2.33±0.41	0.001
PIGD score	4.62±3.18	6.18±3.27	2.43±1.42	3.83±1.32	0.001
Tremor score	7.05±5.85	2.95±2.64	12.86±4.31	8.67±3.61	0.001
Age of motor symptom onset	59.54±9.46	59.16±8.55	60.59±10.80	56.08±8.75	0.474
Montreal Cognitive Assessment	25.32±3.14	25.19±3.28	25.62±3.06	24.50±2.43	0.506
Mini Mental State Exam	28.72±1.7	28.48±1.9	29.12±1.1	28.33±1.0	0.038
Geriatric Depression Scale	4.07±3.35	3.89±2.93	3.83±3.67	7.67±3.56	0.934
Pull test “Off”	1.15±1.25	1.56±1.28	0.52±0.86	1.17±1.47	0.001
Freezing of Gait (FOG-Q)	4.29±7.83	6.27±8.71	1.38±5.32	4.67±8.16	0.001
Number of falls (year prior to the study)	1.91±7.63	3.22±10.11	0.31±0.68	0.21±0.45	0.033

### Clinical Assessment

Parkinsonian symptoms and disease severity were measured using the new version of the UPDRS introduced by Goetz et al. in 2008 [61]. (Note that this new version of the UPDRS contains all of the items of the previous version as well as several new items). Disease duration was defined as time since diagnosis in years. In addition to observation during the entire clinical testing, the new Freezing of Gait questionnaire (FOG-Q) was used to assess if the subject experiences freezing episodes and its severity [62]; in this test, video segments were shown to the subject and then he or she was asked to complete a structured questionnaire that characterizing FOG severity. Subjects walked back and forth along a 35 meter corridor under single and dual task conditions (i.e., while subtracting serial 3s). Gait speed in the off state was determined by measuring the average time the subject walked along the middle 10 meters of a the corridor.

The number of falls experienced one year prior to participation in the study was collected as well via self-report. The cognitive assessment included the Montreal Cognitive Assessment (MoCA) [63] and the Mini Mental State Exam (MMSE) [64]. Since cerebro-vascular risk factors have been associated with and may contribute to WM changes [65], [66], cerebro-vascular risk factors were also assessed (i.e., history of high blood pressure, supine and standing blood pressure, hypercholesterolemia, history of smoking, history of transient ischemic attacks, diabetes). Further, the total number of risk factors was computed as a cerebro-vascular risk index. Deep WMHs have also been associated with altered functioning of the frontostriatal circuits and depressive symptoms in both the general elderly and patients with PD [67], [68] and could, perhaps, be a mediator of gait disturbances in PD. To address this possibility, we also assessed depressive symptoms using the Geriatric Depression Scale [69].

### MRI Acquisition

Acquisition was performed in the “on” medication state using a GE 3T imaging system. All MRI analyses were performed in a blinded manner without knowledge of the subject’s age, gender, UPDRS scores, or group assignment. A fluid attenuated inversion recovery (FLAIR) sequence was used for the semi-quantitative visual rating of WMHs and for the automated methods using the following parameters: TR = 6,200 milliseconds, TE = 200 milliseconds, flip angle = 90, slice thickness = 1.2 mm, matrix = 256×256, FOV = 30.7×30.7 cm^2^.

WMHs were first visually examined and evaluated by a blinded rater using the visual rating of signal hyperintensities, as developed by Scheltens and colleagues [70]. Scheltens’ method quantifies the number, size and location of WM lesions. This scale has good inter- and intra-rater agreement [71] and has been validated in several previous studies[72]–[74]. The Schelten rating scale provides summary scores in a semi-quantitative way: periventricular hyperintensities (PVH scored as 0–6, where 0 indicates no hyperintensities) and lobar white matter (WMH scored as 0–24). Operationally, PVH were identified as continuous confluent areas of high signal intensity adjacent to anterior or posterior horns of the lateral ventricles (“caps”) and along the lateral ventricles (“bands”). WMH, located in the deep and subcortical white matter, were separately rated according to anatomical location (i.e., the frontal, parietal, temporal, and occipital regions) [75]. The manual segmentation of the four lobes was performed using an axial view of a representative volume segmentation for manual tracing of the lobes [76].

Given the inconsistency among previous reports on WMHs in PD[77]–[80], we also used two automated methods to further investigate possible white matter differences between the PIGD and TD groups. One automated method was suggested and validated recently by Smart and colleagues [81]. In brief, the Flair images were first spatially normalized to the T1 weighted MNI (Montreal Neurological Institute) template using SPM (Wellcome Department of Imaging Neuroscience Group, London, UK; http://www.fil.ion.ucl.ac.uk/spm). Following this normalization, images were automatically segmented, based on prior probability maps of the relative distribution of tissue types, into grey matter (GM), white matter (WM) and cerebrospinal fluid (CSF), as implemented in the VBM toolbox in SPM5. WMHs were selected by labeling voxels with intensities greater than 1.45 times the mean WM voxel intensity. Then, the WMHs score was calculated as the ratio between detected WMHs voxels and total WM voxels. For this method, the term WMHref-total will be used.

The second automatic method followed the approach used by Bohnen et al. [82]. The mean intensity of cerebellar white matter voxels on magnetic resonance FLAIR images served as a reference. WMHs were selected by labeling voxels with intensity greater than 1.65 times the mean voxel intensity in the cerebellum. The cerebellum is used as a reference in this method based on the clinical observation that this region is generally spared from age-associated WM changes. For this method, we use the term WMHref-cereb.

### Statistical Analysis

The PIGD and the TD groups were compared using Student’s t-test for continuous variables (or its non-parametric equivalent) and Chi-square tests were used for categorical variables (e.g., gender) to see if the groups were similar with respect to background measures, demographics, and WM scoring. Associations among the different measures were tested using correlation analyses (Pearson or Spearman, as appropriate) and using partial correlations adjusting for potential covariates (e.g., age). All statistical analyses were two-sided and conducted using the Statistical Package for Social Sciences (Version 19 for Windows; SPSS Inc., Chicago, IL, U.S.A.). Average values are reported as mean±standard deviation. Significance was set at the 0.05 level for all analyses.

## Results

### Demographics and Clinical Findings

110 patients with PD participated in this study. Basic characteristics and demographics of all the subjects and the PIGD and TD subtypes are summarized in Table 1. Based on the categorization proposed by Jankovic et al [83], 42 patients (38.2%) were classified as TD, 62 patients (56.4%) were classified as PIGD, and six patients (5.5%) were classified as undetermined. Among all subjects, the PIGD score ranged from 0–14, while the Tremor score ranged from 0–23. As indicated in Table 1, the PIGD and TD subtypes were similar with respect to basic demographics and disease characteristics. As expected, significant group differences in motor symptoms related to PIGD and TD were observed. In general, the subjects were non-demented, as indicated by relatively high scores on the MMSE and MoCA. Interestingly, 48 of the subjects had a MoCA score of 25 or below (with similar means and distributions in both groups) suggesting that many of the subjects had some degree of cognitive decline.

Mean gait speed among all subjects in the off state was 1.11±0.21 m/sec, ranging from 0.49–1.58 m/sec. The mean gait speed in the dual task condition was 0.98±0.26 m/sec, ranging from 0.26–1.52 m/sec. Gait speed was significantly lower (p = 0.049) in the PIGD group (1.08±0.21 m/sec), compared to the TD group (1.17±0.20 m/sec). A marginal effect was found in the dual task condition; walking speed tended (p = 0.088) to be lower in the PIGD patients (0.94±0.28 m/sec), compared to the TD patients (1.04±0.21 m/sec). Five subjects from the PIGD group had more than 3 falls in the year prior to the study. In contrast, none of the TD patients had this many falls. In general, patients from the PIGD subtype had significantly more falls than patients from the TD group (Table 1).

### MRI Findings

From among the 110 patients, MRI data was not fully available for 6 subjects. Four patients were unable to complete the MRI scan due to intolerance to noise or inability to lie still while prone in the magnet. In addition, the first two patients who participated in this protocol did not have the FLAIR sequence needed for the visual rating. Before starting the project, we were concerned that severe tremor might disturb the MRI procedure and impact on the quality of the images. However, this was not the case; for all participants, the quality of the MRI signals was not affected by tremor or dyskinesias. Thus, complete MRI data were obtained in 104 patients.

**Figure 1:**
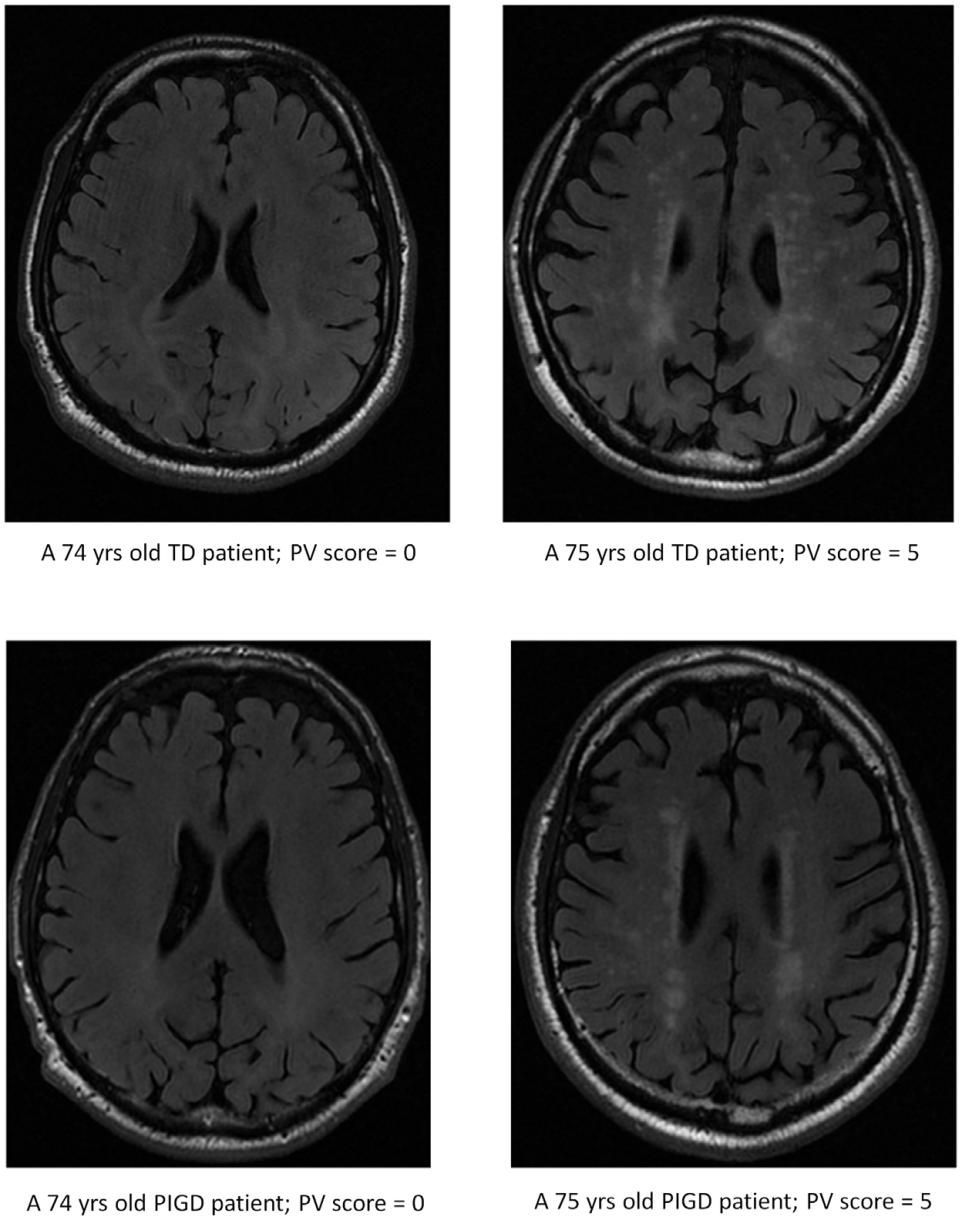
An example of periventricular WMHs in two patients with the PIGD subtype and two patients with the TD subtype. As shown, a subject with PIGD had a low score on the Scheltens scale, while another had a relatively high score (5 out of max. possible 6). Similarly, one patient in the TD group had a low score, while another had a relatively high score. These examples are consistent with the lack of an association between the Scheltens’ scoring and PIGD and TD subtypes that was seen in general.

WMHs results based on Scheltens’ visual rating scale are summarized in Table 2. As indicated, there were no significant PIGD vs. TD group differences in the mean scores or in the percent of subjects with lesions, overall or in specific brain regions. Examples of periventricular WMHs in patients with PIGD and TD are shown in Figure 1. This figure illustrates how the PIGD-TD grouping can be independent of WMHs as scored using Scheltens’ method.

**Table 2:** Visual rating of White Matter Hyperintensities according to Schelten et al.

			Group Comparisons		
	Outcome Measure		PIGD (N = 59) (Mean±SD or n, %)	TD (N = 40) (Mean±SD or n, %)	P-Value PIGD vs. TD
**Deep WMHs**	Frontal	With lesions, n (%)	39 (66.1%)	27 (67.3%)	0.885
		Score, mean± SD	1.83±1.77	1.97±1.95	0.713
	Parietal	With lesions, n (%)	27 (45.8%)	18 (45.2%)	0.940
		Score, mean± SD	1.22±1.72	1.05±1.37	0.613
	Temporal	With lesions, n (%)	10 (16.9%)	6 (15.4%)	0.796
		Score, mean± SD	0.21±0.52	0.27±0.73	0.624
	Occipital	With lesions, n (%)	10 (16.9%)	11 (27.2%)	0.208
		Score, mean± SD	0.43±1.05	0.65±1.38	0.385
	Sum (possible range 0–24)	Score, mean± SD	3.66±3.96	3.95±3.97	0.732
**Periventricular Hyperintensities**	Frontal	With lesions, n (%)	15 (25.4%)	14 (35.9%)	0.352
		Score, mean± SD	0.33±0.63	0.43±0.65	0.446
	Occipital	With lesions, n (%)	16 (27.1%)	11 (28.2%)	0.967
		Score, mean± SD	0.38±0.68	0.41±0.73	0.870
	Bands	With lesions, n (%)	27 (45.8%)	19 (48.7%)	0.865
		Score, mean± SD	0.60±0.75	0.66±0.78	0.707
	Sum (possible range 0–6)	Score, mean± SD	1.31±1.73	1.5±1.69	0.606
	Total sum (DWM+PVH)	Score, mean± SD	4.97±5.39	5.45±5.40	0.678

Deep White Matter Hyperintensities (DWM); Periventricular Hyperintensities (PVH).

**Figure 2:**
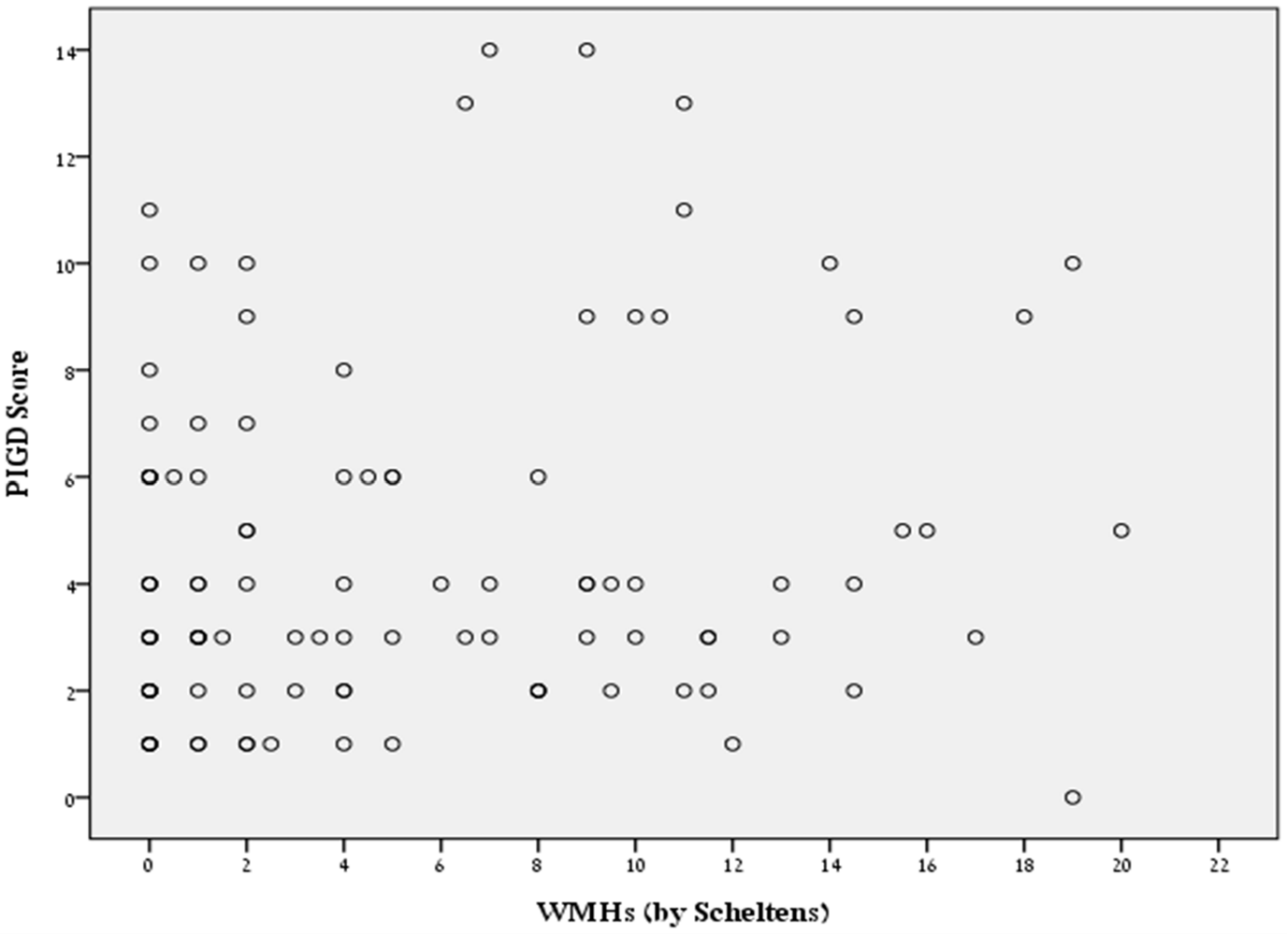
A scatter plot showing the absence of an association between the PIGD score and the total (periventricular and deep) WMHs scores, as determined using the Scheltens methods. WMHs were not correlated with PIGD symptoms.

As expected, across almost all brain regions, significant mild to moderate correlations between WMHs and age were found. Pearson’s correlation coefficients ranged from r = 0.26 to r = 0.41. However, no correlations were found between WMHs and the PIGD scores (Figure 2), TD scores, or disease duration (see Table 3). The absence of correlations between WMHs and PIGD and TD scores persisted in partial correlations that adjusted for age and/or disease duration; correlations between WMHs and the PIGD score (p>0.437) and with the TD score (p>0.179) were not significant. Similarly, correlations between WMHs and the objective continuous measure from the last five items of part 3 of the UPDRS (with or without including the FOG item and the gait sub-scores) were mild (r<0.25) and were no longer significant after adjusting for age.

**Table 3:** Correlations between Schelten’s scoring of WMHs and PIGD and TD symptom.

	Outcome Measure	Age	Disease Duration	PIGD Score	TD Score
**Deep White Matter Hyperintensities**	Frontal	r = 0.40* (0.001)	r = 0.007 (0.941)	r = 0.12 (0.217)	r = 0.06 (0.558)
	Parietal	r = 0.32 (0.001)	r = 0.20 (0.867)	r = 0.16 (0.109)	r = 0.01 (0.923)
	Temporal	r = 0.03 (0.781)	r = 0.03 (0.780)	r = −0.04 (0.685)	r = 0.03 (0.760)
	Occipital	r = 0.23 (0.021)	r = 0.03 (0.735)	r = 0.04 (0.699)	r = 0.12 (0.239)
	Sum DWM	r = 0.38 (0.001)	r = 0.03 (0.731)	r = 0.12 (0.223)	r = 0.06 (0.531)
**Periventricular Hyperintensities**	Frontal	r = 0.39 (0.001)	r = 0.04 (0.698)	r = 0.05 (0.614)	r = 0.16 (0.102)
	Occipital	r = 0.26 (0.008)	r = 0.05 (0.630)	r = 0.12 (0.249)	r = 0.07 (0.486)
	Bands	r = 0.33 (0.001)	r = 0.17 (0.082)	r = 0.16 (0.110)	r = 0.16 (0.103)
	Sum PV	r = 0.41 (0.001)	r = 0.13 (0.180)	r = 0.13 (0.196)	r = 0.17 (0.082)
	Total sum DWM & PV	r = 0.40 (0.001)	r = 0.07 (0.457)	r = 0.12 (0.214)	r = 0.10 (0.337)

* Entries are Pearson’s correlation coefficient and the associated p-value. Deep White Matter Hyperintensities (DWM); Periventricular Hyperintensities (PVH).

We applied Scheltens’ approach specifically to the basal ganglia, the hallmark of PD and the thalamus, one of the likely sources of tremor in PD [84], [85]. Only two patients (one from the PIGD group and one undetermined) had WM lesions in the thalamus, while two other patients had lesions in the basal ganglia. According to this method, WM lesions were not common in the basal ganglia and the thalamus, and did not differ in PD subtypes.

Using the automated method with the cerebellum as a reference (WMHref-cereb), the mean number of voxels with WMHs was similar in the two groups (p = 0.413); the number of voxels with WMHs was 12143.4±20472.8 in the PIGD group and 16004.5±25172.2 in the TD group. In addition, the mean WMHs ratio was 0.0244±0.0382 in the PIGD group and 0.0240±0.0047 in the TD group (p = 0.678). Using the other automated method, WMHref-total, WMHs were also not larger in the PIGD group. In fact, this method suggests that there was a higher WMH burden in the TD group compared to the PIGD group (number of WMH voxels: PIGD 4117.08±4818.02; TD: 7033.26±6963.99; p = 0.027). Similarly, the WMH ratio was significantly higher (p = 0.027) in the TD group (0.0135±0.0132), compared to the PIGD group (0.0079±0.0091), indicating greater WMHs in the TD group, according to the WMHref-total approach. Similar results were obtained for the two automated methods if the results were log transformed.

We further calculated quartiles of the WMHs variables and compared the 1^st^ quartile (fewest WMHs) to the 4^th^ quartile (most WMHs). This analysis, again, did not detect differences between the subtypes. For the WMHref-cereb method, there was no association between the 1^st^ and 4^th^ quartile and PIGD grouping (p = 0.99). For example, among the subjects with the most WMHs, 45% were in the TD group; similarly, among the subjects with the least amount of WMH, 45% were from the TD group. A comparison of the quartiles based on the second automated method (WMHref-total) also revealed no significant association with PIGD and TD grouping (p = 0.217). When we performed the same analysis using the semi-quantitative visual rating scores, again, no significant association with PIGD and TD grouping was found (p = 0.166).

When we applied the more strict classification criteria to assign subjects into representative motor subtypes, 29 (26.4%) patients were included in the predominantly-PIGD subtype (p-PIGD) and 31 (28.2%) patients were assigned to the predominantly-TD (p-TD) subtype; (mean age: 64.9±7.7 vs. 64.6±11.6 yrs, respectively; p = 0.900). Age of motor symptom onset (p = 0.968) and disease duration (p = 0.700) were also similar in both of these groups. Here as well, no significant differences were observed for all WMHs measures. For example, using the visual scoring method, the total score of periventricular hyperintensities was 1.31±1.82 in the p-PIGD group and 1.31±1.61 in the p-TD (p = 0.993). Using the automated method WMHref-cereb, the WMH ratios were 0.0240±0.0031 in the PIGD group and 0.0238±0.0048 in the TD group (p = 0.901).

### Associations between WMHs, Freezing of Gait and Falls

The number of falls in the previous year was moderately correlated with the PIGD score (r = 0.471, p<0.001), but not with the TD score. In addition, a mild correlation was found between the number of falls and the freezing of gait questionnaire score (FOG-Q); (r = 0.309, p = 0.001). However, no significant correlation was found between number of falls and WMHs using all three methods (p>0.471). The FOG-Q score was highly correlated with the PIGD score (r = 0.770, p<0.001), but not with the TD score. The FOG-Q score was not correlated with WMHs using the Scheltens’ score (p>0.287) or with WMHref-cereb scores (p>0.710). Surprisingly, patients with more FOG episodes (i.e., higher FOG-Q scores) tended to have less WMHs using the WMHref-total method (r = −0.172, p = 0.093).

### Association between WMHs and Cerebro-vascular Risk Factors

Table 4 summarizes the cerebro-vascular risk factors among the two groups. Overall, no significant differences were found between the groups (except that more patients in the TD group tended to have diabetes). Moreover, the cerebro-vascular risk index (based on the presence or absence of any risk factor) suggests that, in general, risk factors were similar in the two groups.

**Table 4:** Cerebro-vascular risk factors.

		Group Comparisons		
Outcome Measure	All Patients*	PIGD Patients	TD Patients	P-Value PIGD vs. TD
Diabetes	9.6%	5.0%	17.5%	0.093
High Blood Pressure (by history)	49.5%	45.9%	56.1%	0.546
Supine BP (Systolic) (mmHg)	125.61±15.91	126.95±16.57	124.64±15.18	0.490
Supine BP (Diastolic) (mmHg)	74.74±10.29	75.91±9.47	73.33±11.27	0.228
Years of Smoking	8.57±14.63	10.27±15.56	6.31±13.75	0.213
Hyper-cholestermia	39.3%	45.9%	29.3%	0.241
Vascular Risk Index (possible range: 0–4)	23.1%31.5%29.6%15.7%	17.7%35.5%30.6%16.1%	30.8%23.1%28.2%17.9%	0.595

* Entries are % of subjects or mean±SD.

## Discussion

We hypothesized that greater WM changes would be observed in the PIGD-subtype compared to the TD subtype. The PIGD and TD groups differed with respect to many motor symptoms, as expected. Falls were more common in the patients with PIGD and they also had worse postural control. We could not, however, find any evidence to support our hypothesis. The three different methods that were applied for detecting WMHs showed no increase in WMHs among the PIGD (or even among the p-PIGD) patients, compared to patients with TD. Some visual rating scales may suffer from ceiling effects [72], [86]. Nonetheless, we did not observe this problem and the results from the two automated methods also were not associated with PIGD symptoms. In addition, very mild correlations between WMHs and UPDRS derived measures were observed, however, these correlations were not observed after adjusting for age. Thus, while the association between WMHs, gait, mobility, impaired balance, falls and cognitive deficits are well established in otherwise healthy older adults [87], [88], the present findings do not support this relationship among patients with PD.

In contrast to our findings, Bohnen et al. [89] reported that WMHs burden was significantly related to the PIGD motor phenotype. However, their analysis did not adjust for age, a factor known to be associated with WMHs [90], [91]. In addition, the classification method for dividing the patients into subtypes in that study appears to be slightly different from that originally proposed by Jankovic et al [92]. Moreover, while leukoaraiosis was significantly correlated with posture and postural stability by examination and with walking by history, it was only marginally correlated with freezing of gait and no correlation was found with falls in the study by Bohnen et al. Using conventional MRI obtained with a 1.5-T system, Lee et al. [93] observed greater WMHs in the PIGD patients compared to TD, graded according to the atherosclerosis risk in communities (ARIC) study, another visual rating scale. These methodological differences may explain the differences between our findings and those of Bohnen et al. and Lee et al., however, future studies are needed to resolve these somewhat puzzling and conflicting results.

In a recent systemic review about WMHs, physical functioning and falls in older adults, Zheng at al. [94] concluded that impaired mobility and an increased fall risk are only evident in people who have the most severe degree of WMHs. We tested this possibility when we examined the relationship between PIGD grouping and WMH quartiles. Even after stratifying the WMHs variables into quartiles to compare only patients with least and most severe degrees of WM changes, we failed to find an association between WMHs and PIGD symptoms. These findings also suggest that the relationships observed in older adults are not found in patients with PD, even among those with relatively severe leukoaraiosis. Perhaps impairments due to dopamine loss make the WMHs less relevant among patients with PD.

The mechanisms linking WMHs and motor function in older adults are not fully understood. However, reports on the association of frontal and periventricular age-related WM changes with falls support the hypothesis that interruption of frontal subcortical motor circuits lead to balance disturbances and hence to an increased risk for falls [95]. A recent review of studies among older adults supports the idea that a high WM burden is associated with gait disorders and observed that the largest WMH fractions were found in the frontal lobe, the centrum semiovale, the posterior limb of internal capsule, the genu and the splenium of corpus callosum [96]. In the present study, two thirds of the subjects had WMHs in frontal regions, while lesions were less common in other regions (recall Table 3). Still, there was no association between lesion location and gait disturbances, further suggesting that the relationship between WMHs and gait and falls in patients with PD is different from that observed in older adults who do not have PD.

If WMHs do not play a major role in the PIGD clinical phenotype, other pathological processes must explain the existence of the motor subtypes in PD. One possible explanation for the differences between PIGD and TD might be due to gray matter (GM) alterations. Tremor has been related to GM atrophy in the thalamus [97] and in the cerebellum [98]. Additionally, in a recent study by Kostic et al. [99], PD patients with freezing of gait showed a distributed pattern of GM atrophy compared to controls and compared to PD patients without FOG, reinforcing the possible association between axial symptoms and GM frontal and parietal atrophy. Indirectly, genetics might also contribute to the different motor symptoms, although there have been conflicting reports in the literature regarding the link between mutations and the clinical subtypes [100], [101] ^,^ [102]. A third possibility is that the group differences may be related to more subtle changes in WM. In the present study, our focus was on leukoaraiosis; perhaps more advanced technology (e.g., 7 Tesla MRI) or imaging techniques that examine more subtle changes in WM organization and the alignment of fibers (e.g., diffusion tensor imaging, DTI) [103], [104] might provide some of the explanation. Another possibility is that the anti-parkinsonian medications that are aimed at treating motor symptoms in PD may alter the expression of WMHs. Finally, it is possible that distinct patterns of neurodegeneration contribute to the different clinical features. One can speculate that pathology at the level of the brainstem nuclei could affect cerebellar outflow as well as the degree of involvement of the pedunculo-pontine nucleus (PPN) or the nucleus Basalis of Meinert. As a result, differences between a PIGD course or TD course could be the result of different functional connectivity between those centers causing differentiation at the level of brain networks. These questions can only be answered by large scale clinical-pathological studies that are augmented by functional imaging methods.

### Strengths, Limitations and Future Directions

Strengths of the present study include the relatively large sample and the use of three different methods for evaluation and analysis of WMHs, all performed while blinded to PIGD and TD group assignments. One limitation of this study is its cross-sectional nature which precludes the investigation of changes over time. In the future, prospective documentation of natural history of PD and its association (or lack thereof) with WMHs, disease progression, changes in the clinical phenotypes, vascular risk factors, and markers of inflammation may shed additional light on the pathogenesis of the PIGD motor subtype. In addition, it would be helpful to compare each of the subtypes to an age-matched control group in the future. Another limitation is the exclusion of subjects with significant gait impairment in the off medication state and patients with mild to moderate dementia. These criteria might have biased the study and may have lead to the negative association. Nonetheless, we note that there was a wide range in both motor and cognitive abilities (recall Table 1).

### Conclusions

To improve a clinician’s ability to tailor therapy to the individual patient, it is important to understand why a subset of patients with PD primarily have PIGD symptoms, while others experience mainly tremor with relatively little gait and postural difficulties. In contrast to our expectations, the present findings suggest that cererbro-vascular findings and white matter structural changes are likely not the cause of these differences. Future studies should, therefore, focus their efforts on other possible explanations.

## References

[1] Jankovic J (2008) Parkinson’s disease: clinical features and diagnosis. J Neurol Neurosurg Psychiatry 79: 368–76. 18344392 10.1136/jnnp.2007.131045

[2] Jankovic J, McDermott M, Carter J, Gauthier S, Goetz C, et al (1990) Variable expression of Parkinson’s disease: a base-line analysis of the DATATOP cohort. The Parkinson Study Group. Neurology 40: 1529–34. 2215943 10.1212/wnl.40.10.1529

[3] van Rooden SM, Colas F, Martinez-Martin P, Visser M, Verbaan D, et al (2011) Clinical subtypes of Parkinson’s disease. Mov Disord 26: 51–8. 21322019 10.1002/mds.23346

[4] Selikhova M, Williams DR, Kempster PA, Holton JL, Revesz T, et al (2009) A clinico-pathological study of subtypes in Parkinson’s disease. Brain 132: 2947–57. 19759203 10.1093/brain/awp234

[5] Murray ME, Senjem ML, Petersen RC, Hollman JH, Preboske GM, et al (2010) Functional impact of white matter hyperintensities in cognitively normal elderly subjects. Arch Neurol 67: 1379–85. 21060015 10.1001/archneurol.2010.280 PMC3025610

[6] Benson RR, Guttmann CR, Wei X, Warfield SK, Hall C, et al (2002) Older people with impaired mobility have specific loci of periventricular abnormality on MRI. Neurology 58: 48–55. 11781405 10.1212/wnl.58.1.48

[7] Soumare A, Elbaz A, Zhu Y, Maillard P, Crivello F, et al (2009) White matter lesions volume and motor performances in the elderly. Ann Neurol 65: 706–15. 19557865 10.1002/ana.21674

[8] Baezner H, Blahak C, Poggesi A, Pantoni L, Inzitari D, et al (2008) Association of gait and balance disorders with age-related white matter changes: the LADIS study. Neurology 70: 935–42. 18347315 10.1212/01.wnl.0000305959.46197.e6

[9] Blahak C, Baezner H, Pantoni L, Poggesi A, Chabriat H, et al (2009) Deep frontal and periventricular age related white matter changes but not basal ganglia and infratentorial hyperintensities are associated with falls: cross sectional results from the LADIS study. J Neurol Neurosurg Psychiatry 80: 608–13. 19204027 10.1136/jnnp.2008.154633

[10] Franch O, Calandre L, Alvarez-Linera J, Louis ED, Bermejo-Pareja F, et al (2009) Gait disorders of unknown cause in the elderly: Clinical and MRI findings. J Neurol Sci 280: 84–6. 19251276 10.1016/j.jns.2009.02.001

[11] Novak V, Haertle M, Zhao P, Hu K, Munshi M, et al (2009) White matter hyperintensities and dynamics of postural control. Magn Reson Imaging 27: 752–9. 19250785 10.1016/j.mri.2009.01.010 PMC2727871

[12] Wolfson L, Wei X, Hall CB, Panzer V, Wakefield D, et al (2005) Accrual of MRI white matter abnormalities in elderly with normal and impaired mobility. J Neurol Sci 232: 23–7. 15850578 10.1016/j.jns.2004.12.017

[13] de Laat KF, Tuladhar AM, van Norden AG, Norris DG, Zwiers MP, et al (2011) Loss of white matter integrity is associated with gait disorders in cerebral small vessel disease. Brain 134: 73–83. 21156660 10.1093/brain/awq343

[14] Baloh RW, Vinters HV (1995) White matter lesions and disequilibrium in older people. II. Clinicopathologic correlation. Arch Neurol 52: 975–81. 7575225 10.1001/archneur.1995.00540340067014

[15] Bohnen NI, Albin RL (2011) White matter lesions in Parkinson disease. Nat Rev Neurol 7: 229–36. 21343896 10.1038/nrneurol.2011.21 PMC3739056

[16] Gunning-Dixon FM, Raz N (2000) The cognitive correlates of white matter abnormalities in normal aging: a quantitative review. Neuropsychology 14: 224–32. 10791862 10.1037//0894-4105.14.2.224

[17] Hedden T, Van Dijk KR, Shire EH, Sperling RA, Johnson KA et al.. (2011) Failure to Modulate Attentional Control in Advanced Aging Linked to White Matter Pathology. Cereb Cortex. 10.1093/cercor/bhr172 PMC3328340 21765181

[18] Silbert LC, Nelson C, Howieson DB, Moore MM, Kaye JA (2008) Impact of white matter hyperintensity volume progression on rate of cognitive and motor decline. Neurology 71: 108–13. 18606964 10.1212/01.wnl.0000316799.86917.37 PMC2676966

[19] Prins ND, van Dijk EJ, den HT, Vermeer SE, Jolles J, et al (2005) Cerebral small-vessel disease and decline in information processing speed, executive function and memory. Brain 128: 2034–41. 15947059 10.1093/brain/awh553

[20] Tullberg M, Fletcher E, DeCarli C, Mungas D, Reed BR, et al (2004) White matter lesions impair frontal lobe function regardless of their location. Neurology 63: 246–53. 15277616 10.1212/01.wnl.0000130530.55104.b5 PMC1893004

[21] Dubois B, Pillon B (1997) Cognitive deficits in Parkinson’s disease. J Neurol 244: 2–8. 9007738 10.1007/pl00007725

[22] Verbaan D, Marinus J, Visser M, van Rooden SM, Stiggelbout AM, et al (2007) Cognitive impairment in Parkinson’s disease 14. J Neurol Neurosurg Psychiatry 78: 1182–7. 17442759 10.1136/jnnp.2006.112367 PMC2117586

[23] Cameron IG, Watanabe M, Pari G, Munoz DP (2010) Executive impairment in Parkinson’s disease: response automaticity and task switching. Neuropsychologia 48: 1948–57. 20303998 10.1016/j.neuropsychologia.2010.03.015

[24] Hausdorff JM, Doniger GM, Springer S, Yogev G, Giladi N, et al (2006) A common cognitive profile in elderly fallers and in patients with Parkinson’s disease: the prominence of impaired executive function and attention. Exp Aging Res 32: 411–29. 16982571 10.1080/03610730600875817 PMC1868891

[25] Beyer MK, Aarsland D, Greve OJ, Larsen JP (2006) Visual rating of white matter hyperintensities in Parkinson’s disease. Mov Disord 21: 223–9. 16161159 10.1002/mds.20704

[26] Beyer MK, Janvin CC, Larsen JP, Aarsland D (2007) A magnetic resonance imaging study of patients with Parkinson’s disease with mild cognitive impairment and dementia using voxel-based morphometry. J Neurol Neurosurg Psychiatry 78: 254–9. 17028119 10.1136/jnnp.2006.093849 PMC2117633

[27] Bohnen NI, Albin RL (2011) White matter lesions in Parkinson disease. Nat Rev Neurol 7: 229–36. 21343896 10.1038/nrneurol.2011.21 PMC3739056

[28] Choi SA, Evidente VG, Caviness JN, Shill HA, Sabbagh MN, et al (2010) Are there differences in cerebral white matter lesion burdens between Parkinson’s disease patients with or without dementia? Acta Neuropathol 119: 147–9. 19956959 10.1007/s00401-009-0620-2 PMC2928051

[29] Slawek J, Wieczorek D, Derejko M, Dubaniewicz M, Brockhuis B, et al (2008) The influence of vascular risk factors and white matter hyperintensities on the degree of cognitive impairment in Parkinson’s disease. Neurol Neurochir Pol 42: 505–12. 19235103

[30] Slawek J, Roszmann A, Robowski P, Dubaniewicz M, Sitek EJ et al.. (2012) The Impact of MRI White Matter Hyperintensities on Dementia in Parkinson’s Disease in Relation to the Homocysteine Level and Other Vascular Risk Factors. Neurodegener Dis. 10.1159/000338610 22831964

[31] Green J, McDonald WM, Vitek JL, Evatt M, Freeman A, et al (2002) Cognitive impairments in advanced PD without dementia. Neurology 59: 1320–4. 12427877 10.1212/01.wnl.0000031426.21683.e2

[32] Rodriguez-Ferreiro J, Cuetos F, Herrera E, Menendez M, Ribacoba R (2010) Cognitive impairment in Parkinson’s disease without dementia. Mov Disord 25: 2136–41. 20725913 10.1002/mds.23239

[33] Giladi N, Herman T, Reider-Groswasser II, Gurevich T, Hausdorff JM (2005) Clinical characteristics of elderly patients with a cautious gait of unknown origin. J Neurol 252: 300–6. 15726273 10.1007/s00415-005-0641-2

[34] Giladi N, Huber-Mahlin V, Herman T, Hausdorff JM (2007) Freezing of gait in older adults with high level gait disorders: association with impaired executive function. J Neural Transm 114: 1349–53. 17576512 10.1007/s00702-007-0772-y

[35] Gurevich T, Giladi N (2003) Freezing of gait in multiple system atrophy (MSA). Parkinsonism Relat Disord 9: 169–74. 12573873 10.1016/s1353-8020(02)00049-4

[36] Herman T, Giladi N, Gurevich T, Hausdorff JM (2005) Gait instability and fractal dynamics of older adults with a “cautious” gait: why do certain older adults walk fearfully? Gait Posture 21: 178–85. 15639397 10.1016/j.gaitpost.2004.01.014

[37] Huber-Mahlin V, Giladi N, Herman T, Perez C, Gurevich T, et al (2010) Progressive nature of a higher level gait disorder: a 3-year prospective study. J Neurol 257: 1279–86. 20204394 10.1007/s00415-010-5507-6

[38] Stolze H, Kuhtz-Buschbeck JP, Drucke H, Johnk K, Illert M, et al (2001) Comparative analysis of the gait disorder of normal pressure hydrocephalus and Parkinson’s disease. J Neurol Neurosurg Psychiatry 70: 289–97. 11181848 10.1136/jnnp.70.3.289 PMC1737236

[39] Poggesi A, Pracucci G, Chabriat H, Erkinjuntti T, Fazekas F, et al (2008) Urinary complaints in nondisabled elderly people with age-related white matter changes: the Leukoaraiosis And DISability (LADIS) Study. J Am Geriatr Soc 56: 1638–43. 18691285 10.1111/j.1532-5415.2008.01832.x

[40] Verbaan D, Marinus J, Visser M, van Rooden SM, Stiggelbout AM, et al (2007) Cognitive impairment in Parkinson’s disease 14. J Neurol Neurosurg Psychiatry 78: 1182–7. 17442759 10.1136/jnnp.2006.112367 PMC2117586

[41] Verbaan D, Marinus J, Visser M, van Rooden SM, Stiggelbout AM, et al (2007) Patient-reported autonomic symptoms in Parkinson disease. Neurology 69: 333–41. 17646625 10.1212/01.wnl.0000266593.50534.e8

[42] Bohnen NI, Muller ML, Zarzhevsky N, Koeppe RA, Bogan CW, et al (2011) Leucoaraiosis, nigrostriatal denervation and motor symptoms in Parkinson’s disease. Brain 134: 2358–65. 21653540 10.1093/brain/awr139 PMC3155702

[43] Piccini P, Pavese N, Canapicchi R, Paoli C, Del DP, et al (1995) White matter hyperintensities in Parkinson’s disease. Clinical correlations. Arch Neurol 52: 191–4. 7848130 10.1001/archneur.1995.00540260097023

[44] Stern MB, Braffman BH, Skolnick BE, Hurtig HI, Grossman RI (1989) Magnetic resonance imaging in Parkinson’s disease and parkinsonian syndromes. Neurology 39: 1524–6. 2812334 10.1212/wnl.39.11.1524

[45] Acharya HJ, Bouchard TP, Emery DJ, Camicioli RM (2007) Axial signs and magnetic resonance imaging correlates in Parkinson’s disease. Can J Neurol Sci 34: 56–61. 17352348 10.1017/s0317167100005795

[46] Dalaker TO, Larsen JP, Bergsland N, Beyer MK, Alves G, et al (2009) Brain atrophy and white matter hyperintensities in early Parkinson’s disease(a). Mov Disord 24: 2233–41. 19768730 10.1002/mds.22754

[47] Piccini P, Pavese N, Canapicchi R, Paoli C, Del DP, et al (1995) White matter hyperintensities in Parkinson’s disease. Clinical correlations. Arch Neurol 52: 191–4. 7848130 10.1001/archneur.1995.00540260097023

[48] Stern MB, Braffman BH, Skolnick BE, Hurtig HI, Grossman RI (1989) Magnetic resonance imaging in Parkinson’s disease and parkinsonian syndromes. Neurology 39: 1524–6. 2812334 10.1212/wnl.39.11.1524

[49] Acharya HJ, Bouchard TP, Emery DJ, Camicioli RM (2007) Axial signs and magnetic resonance imaging correlates in Parkinson’s disease. Can J Neurol Sci 34: 56–61. 17352348 10.1017/s0317167100005795

[50] Dalaker TO, Larsen JP, Bergsland N, Beyer MK, Alves G, et al (2009) Brain atrophy and white matter hyperintensities in early Parkinson’s disease(a). Mov Disord 24: 2233–41. 19768730 10.1002/mds.22754

[51] Lee SJ, Kim JS, Lee KS, An JY, Kim W, et al (2009) The severity of leukoaraiosis correlates with the clinical phenotype of Parkinson’s disease. Arch Gerontol Geriatr 49: 255–9. 18977043 10.1016/j.archger.2008.09.005

[52] Bohnen NI, Muller ML, Zarzhevsky N, Koeppe RA, Bogan CW, et al (2011) Leucoaraiosis, nigrostriatal denervation and motor symptoms in Parkinson’s disease. Brain 134: 2358–65. 21653540 10.1093/brain/awr139 PMC3155702

[53] Hughes AJ, Daniel SE, Kilford L, Lees AJ (1992) Accuracy of clinical diagnosis of idiopathic Parkinson’s disease: a clinico-pathological study of 100 cases. J Neurol Neurosurg Psychiatry 55: 181–4. 1564476 10.1136/jnnp.55.3.181 PMC1014720

[54] Jankovic J, McDermott M, Carter J, Gauthier S, Goetz C, et al (1990) Variable expression of Parkinson’s disease: a base-line analysis of the DATATOP cohort. The Parkinson Study Group. Neurology 40: 1529–34. 2215943 10.1212/wnl.40.10.1529

[55] Fahn S (1987) Unified Parkinson’s disease rating scale. In Fahn S, Marsden CD, Calne D, Goldstein M, editors. Recent developments in Parkinson’s disease. Florham Park, NJ : Macmillan Health Care Information. 153–63.

[56] Jankovic J, McDermott M, Carter J, Gauthier S, Goetz C, et al (1990) Variable expression of Parkinson’s disease: a base-line analysis of the DATATOP cohort. The Parkinson Study Group. Neurology 40: 1529–34. 2215943 10.1212/wnl.40.10.1529

[57] Jankovic J, McDermott M, Carter J, Gauthier S, Goetz C, et al (1990) Variable expression of Parkinson’s disease: a base-line analysis of the DATATOP cohort. The Parkinson Study Group. Neurology 40: 1529–34. 2215943 10.1212/wnl.40.10.1529

[58] Jankovic J, McDermott M, Carter J, Gauthier S, Goetz C, et al (1990) Variable expression of Parkinson’s disease: a base-line analysis of the DATATOP cohort. The Parkinson Study Group. Neurology 40: 1529–34. 2215943 10.1212/wnl.40.10.1529

[59] Jankovic J, McDermott M, Carter J, Gauthier S, Goetz C, et al (1990) Variable expression of Parkinson’s disease: a base-line analysis of the DATATOP cohort. The Parkinson Study Group. Neurology 40: 1529–34. 2215943 10.1212/wnl.40.10.1529

[60] Jankovic J, McDermott M, Carter J, Gauthier S, Goetz C, et al (1990) Variable expression of Parkinson’s disease: a base-line analysis of the DATATOP cohort. The Parkinson Study Group. Neurology 40: 1529–34. 2215943 10.1212/wnl.40.10.1529

[61] Goetz CG, Tilley BC, Shaftman SR, Stebbins GT, Fahn S, et al (2008) Movement Disorder Society-sponsored revision of the Unified Parkinson’s Disease Rating Scale (MDS-UPDRS): scale presentation and clinimetric testing results. Mov Disord 23: 2129–70. 19025984 10.1002/mds.22340

[62] Nieuwboer A, Rochester L, Herman T, Vandenberghe W, Emil GE et al.. (2009) Reliability of the new freezing of gait questionnaire: Agreement between patients with Parkinson’s disease and their carers. Gait Posture. 10.1016/j.gaitpost.2009.07.108 19660949

[63] Nasreddine ZS, Phillips NA, Bedirian V, Charbonneau S, Whitehead V, et al (2005) The Montreal Cognitive Assessment, MoCA: a brief screening tool for mild cognitive impairment. J Am Geriatr Soc 53: 695–9. 15817019 10.1111/j.1532-5415.2005.53221.x

[64] Folstein MF, Folstein SE, McHugh PR (1975) “Mini-mental state”. A practical method for grading the cognitive state of patients for the clinician. J Psychiatr Res 12: 189–98. 1202204 10.1016/0022-3956(75)90026-6

[65] de Laat KF, Tuladhar AM, van Norden AG, Norris DG, Zwiers MP, et al (2011) Loss of white matter integrity is associated with gait disorders in cerebral small vessel disease. Brain 134: 73–83. 21156660 10.1093/brain/awq343

[66] Silbert LC, Nelson C, Howieson DB, Moore MM, Kaye JA (2008) Impact of white matter hyperintensity volume progression on rate of cognitive and motor decline. Neurology 71: 108–13. 18606964 10.1212/01.wnl.0000316799.86917.37 PMC2676966

[67] Petrovic IN, Stefanova E, Kozic D, Semnic R, Markovic V, Daragasevic N, Kostic V. White matter lesions and depression in patients with Parkinson’s disease. In 2012. 10.1016/j.jns.2012.07.021 22857990

[68] Sneed JR, Culang-Reinlieb ME (2011) The vascular depression hypothesis: an update. Am J Geriatr Psychiatry 19: 99–103. 21328801 10.1097/jgp.0b013e318202fc8a PMC3080245

[69] Yesavage JA, Brink TL, Rose TL, Lum O, Huang V, et al (1982) Development and validation of a geriatric depression screening scale: a preliminary report. J Psychiatr Res 17: 37–49. 7183759 10.1016/0022-3956(82)90033-4

[70] Scheltens P, Barkhof F, Leys D, Pruvo JP, Nauta JJ, et al (1993) A semiquantative rating scale for the assessment of signal hyperintensities on magnetic resonance imaging. J Neurol Sci 114: 7–12. 8433101 10.1016/0022-510x(93)90041-v

[71] Kapeller P, Barber R, Vermeulen RJ, Ader H, Scheltens P, et al (2003) Visual rating of age-related white matter changes on magnetic resonance imaging: scale comparison, interrater agreement, and correlations with quantitative measurements. Stroke 34: 441–5. 12574557 10.1161/01.str.0000049766.26453.e9

[72] Gouw AA, van der Flier WM, van Straaten EC, Pantoni L, Bastos-Leite AJ, et al (2008) Reliability and sensitivity of visual scales versus volumetry for evaluating white matter hyperintensity progression. Cerebrovasc Dis 25: 247–53. 18216467 10.1159/000113863

[73] van Straaten EC, Fazekas F, Rostrup E, Scheltens P, Schmidt R, et al (2006) Impact of white matter hyperintensities scoring method on correlations with clinical data: the LADIS study. Stroke 37: 836–40. 16439704 10.1161/01.STR.0000202585.26325.74

[74] Beyer MK, Aarsland D, Greve OJ, Larsen JP (2006) Visual rating of white matter hyperintensities in Parkinson’s disease. Mov Disord 21: 223–9. 16161159 10.1002/mds.20704

[75] Scheltens P, Barkhof F, Leys D, Pruvo JP, Nauta JJ, et al (1993) A semiquantative rating scale for the assessment of signal hyperintensities on magnetic resonance imaging. J Neurol Sci 114: 7–12. 8433101 10.1016/0022-510x(93)90041-v

[76] Bokde AL, Teipel SJ, Schwarz R, Leinsinger G, Buerger K, et al (2005) Reliable manual segmentation of the frontal, parietal, temporal, and occipital lobes on magnetic resonance images of healthy subjects. Brain Res Brain Res Protoc 14: 135–45. 15795167 10.1016/j.brainresprot.2004.10.001

[77] Bohnen NI, Muller ML, Zarzhevsky N, Koeppe RA, Bogan CW, et al (2011) Leucoaraiosis, nigrostriatal denervation and motor symptoms in Parkinson’s disease. Brain 134: 2358–65. 21653540 10.1093/brain/awr139 PMC3155702

[78] Lee SJ, Kim JS, Lee KS, An JY, Kim W, et al (2009) The severity of leukoaraiosis correlates with the clinical phenotype of Parkinson’s disease. Arch Gerontol Geriatr 49: 255–9. 18977043 10.1016/j.archger.2008.09.005

[79] Acharya HJ, Bouchard TP, Emery DJ, Camicioli RM (2007) Axial signs and magnetic resonance imaging correlates in Parkinson’s disease. Can J Neurol Sci 34: 56–61. 17352348 10.1017/s0317167100005795

[80] Dalaker TO, Larsen JP, Bergsland N, Beyer MK, Alves G, et al (2009) Brain atrophy and white matter hyperintensities in early Parkinson’s disease(a). Mov Disord 24: 2233–41. 19768730 10.1002/mds.22754

[81] Smart SD, Firbank MJ, O’Brien JT (2011) Validation of automated white matter hyperintensity segmentation. J Aging Res 2011: 391783. 21904678 10.4061/2011/391783 PMC3167190

[82] Bohnen NI, Muller ML, Zarzhevsky N, Koeppe RA, Bogan CW, et al (2011) Leucoaraiosis, nigrostriatal denervation and motor symptoms in Parkinson’s disease. Brain 134: 2358–65. 21653540 10.1093/brain/awr139 PMC3155702

[83] Jankovic J, McDermott M, Carter J, Gauthier S, Goetz C, et al (1990) Variable expression of Parkinson’s disease: a base-line analysis of the DATATOP cohort. The Parkinson Study Group. Neurology 40: 1529–34. 2215943 10.1212/wnl.40.10.1529

[84] Kassubek J, Juengling FD, Hellwig B, Spreer J, Lucking CH (2002) Thalamic gray matter changes in unilateral Parkinsonian resting tremor: a voxel-based morphometric analysis of 3-dimensional magnetic resonance imaging. Neurosci Lett 323: 29–32. 11911983 10.1016/s0304-3940(02)00111-8

[85] Zaidel A, Arkadir D, Israel Z, Bergman H (2009) Akineto-rigid vs. tremor syndromes in Parkinsonism. Curr Opin Neurol 22: 387–93. 19494773 10.1097/WCO.0b013e32832d9d67

[86] van Straaten EC, Fazekas F, Rostrup E, Scheltens P, Schmidt R, et al (2006) Impact of white matter hyperintensities scoring method on correlations with clinical data: the LADIS study. Stroke 37: 836–40. 16439704 10.1161/01.STR.0000202585.26325.74

[87] Zheng JJ, Delbaere K, Close JC, Sachdev PS, Wen W et al.. (2011) White matter hyperintensities and impaired choice stepping reaction time in older people. Neurobiol Aging. 10.1016/j.neurobiolaging.2010.12.009 21257231

[88] Zheng JJ, Delbaere K, Close JC, Sachdev PS, Lord SR (2011) Impact of white matter lesions on physical functioning and fall risk in older people: a systematic review. Stroke 42: 2086–90. 21636821 10.1161/STROKEAHA.110.610360

[89] Bohnen NI, Muller ML, Zarzhevsky N, Koeppe RA, Bogan CW, et al (2011) Leucoaraiosis, nigrostriatal denervation and motor symptoms in Parkinson’s disease. Brain 134: 2358–65. 21653540 10.1093/brain/awr139 PMC3155702

[90] de Leeuw FE, de Groot JC, Achten E, Oudkerk M, Ramos LM, et al (2001) Prevalence of cerebral white matter lesions in elderly people: a population based magnetic resonance imaging study. The Rotterdam Scan Study. J Neurol Neurosurg Psychiatry 70: 9–14. 11118240 10.1136/jnnp.70.1.9 PMC1763449

[91] Pantoni L, Poggesi A, Basile AM, Pracucci G, Barkhof F, et al (2006) Leukoaraiosis predicts hidden global functioning impairment in nondisabled older people: the LADIS (Leukoaraiosis and Disability in the Elderly) Study. J Am Geriatr Soc 54: 1095–101. 16866681 10.1111/j.1532-5415.2006.00798.x

[92] Jankovic J, McDermott M, Carter J, Gauthier S, Goetz C, et al (1990) Variable expression of Parkinson’s disease: a base-line analysis of the DATATOP cohort. The Parkinson Study Group. Neurology 40: 1529–34. 2215943 10.1212/wnl.40.10.1529

[93] Lee SJ, Kim JS, Lee KS, An JY, Kim W, et al (2009) The severity of leukoaraiosis correlates with the clinical phenotype of Parkinson’s disease. Arch Gerontol Geriatr 49: 255–9. 18977043 10.1016/j.archger.2008.09.005

[94] Zheng JJ, Delbaere K, Close JC, Sachdev PS, Lord SR (2011) Impact of white matter lesions on physical functioning and fall risk in older people: a systematic review. Stroke 42: 2086–90. 21636821 10.1161/STROKEAHA.110.610360

[95] Blahak C, Baezner H, Pantoni L, Poggesi A, Chabriat H, et al (2009) Deep frontal and periventricular age related white matter changes but not basal ganglia and infratentorial hyperintensities are associated with falls: cross sectional results from the LADIS study. J Neurol Neurosurg Psychiatry 80: 608–13. 19204027 10.1136/jnnp.2008.154633

[96] Annweiler C, Montero-Odasso M (2012) Vascular burden as a substrate for higher-level gait disorders in older adults. A review of brain mapping literature. Panminerva Med 54: 189–204. 22801436

[97] Kassubek J, Juengling FD, Hellwig B, Spreer J, Lucking CH (2002) Thalamic gray matter changes in unilateral Parkinsonian resting tremor: a voxel-based morphometric analysis of 3-dimensional magnetic resonance imaging. Neurosci Lett 323: 29–32. 11911983 10.1016/s0304-3940(02)00111-8

[98] Benninger DH, Thees S, Kollias SS, Bassetti CL, Waldvogel D (2009) Morphological differences in Parkinson’s disease with and without rest tremor. J Neurol 256: 256–63. 19219572 10.1007/s00415-009-0092-2

[99] Kostic VS, Agosta F, Pievani M, Stefanova E, Jecmenica-Lukic M, et al (2012) Pattern of brain tissue loss associated with freezing of gait in Parkinson disease. Neurology 78: 409–16. 22282641 10.1212/WNL.0b013e318245d23c

[100] Healy DG, Falchi M, O’Sullivan SS, Bonifati V, Durr A, et al (2008) Phenotype, genotype, and worldwide genetic penetrance of LRRK2-associated Parkinson’s disease: a case-control study. Lancet Neurol 7: 583–90. 18539534 10.1016/S1474-4422(08)70117-0 PMC2832754

[101] Marras C, Schule B, Munhoz RP, Rogaeva E, Langston JW, et al (2011) Phenotype in parkinsonian and nonparkinsonian LRRK2 G2019S mutation carriers. Neurology 77: 325–33. 21753163 10.1212/WNL.0b013e318227042d PMC3140802

[102] Alcalay RN, Mejia-Santana H, Tang MX, Rosado L, Verbitsky M, et al (2009) Motor phenotype of LRRK2 G2019S carriers in early-onset Parkinson disease. Arch Neurol 66: 1517–22. 20008657 10.1001/archneurol.2009.267 PMC2837584

[103] Vaillancourt DE, Spraker MB, Prodoehl J, Abraham I, Corcos DM, et al (2009) High-resolution diffusion tensor imaging in the substantia nigra of de novo Parkinson disease. Neurology 72: 1378–84. 19129507 10.1212/01.wnl.0000340982.01727.6e PMC2677508

[104] Gattellaro G, Minati L, Grisoli M, Mariani C, Carella F, et al (2009) White matter involvement in idiopathic Parkinson disease: a diffusion tensor imaging study. AJNR Am J Neuroradiol 30: 1222–6. 19342541 10.3174/ajnr.A1556 PMC7051338

